# Evaluation of alveolar bone width alterations around dental implants

**DOI:** 10.6026/973206300200579

**Published:** 2024-05-31

**Authors:** K Sai Priyanka, Pushkar Gupta, Lipika Gopal, A. Karan Kumar, Anas Abdul A. Karan, Banashree Baishya, Nazargi Mohabob

**Affiliations:** 1 Periodontist, Hyderabad, Telangana, India; 2 Department of Prosthodontics and Crown & Bridge , Hitkarini Dental College and Hospital, Jabalpur, MP, India; 3 Department of Periodontolgy, Manav Rachana Dental College, Faridabad, Haryana, India; 4 Department of Prosthodontics and Implantology, Malla Reddy Institute of Dental Sciences, Hyderabad, Telangana, India; 5 Department of Periodontology and Implant Dentistry, College of Dentistry, Qassim University, Kingdom of Saudi Arabia; 6 Department of Periodontics and Oral Implantology, Hi Tech Dental College & Hospital, Bhubaneswar, Odisha, India; 7 Department of Oral Maxillofacial Surgery and Diagnostic Sciences, College of Dentistry, King Faisal University, Al Hofuf Kingdom of Saudi Arabia

**Keywords:** Bone width, CBCT, evaluation, Dental implant

## Abstract

Teeth that are lost can be replaced with dental implants. A sufficient width of bone surrounding the implant is beneficial to its success. Therefore, it is of interest to examine alterations in width of alveolar bone surrounding dental implants at natural and rebuilt bone locations [alveolar ridge preservation (ARP) /Guided Bone Regeneration (GBR)] using CTBT. A CBCT examination of the implant recipient site was performed on sixty patients (both male and female), who had undergone dental implants. All conventional surgical procedures were followed for inserting dental implants. All participants had their horizontal alveolar bone widths around implants assessed at 3 positions: subcrestal width 1 mm (CW1 (crestal level-CW1), subcrestal width 4 mm (CW4), and subcrestal width 7 mm (CW7). There were 32 male patients and 28 female patients out of 60 totals. The mean bone width was 7.02 mm at CW1 prior to surgery and 6.91 mm afterward; it was 8.52 mm at CW4 and 8.13 mm afterward; and it was 10.21 mm at CW7 prior to surgery and 10.08 mm afterward. There was a substantial difference (P<0.05). At CW1, the bone width was 0.38 mm at local bone and -0.02 mm at ARP/GBR; at CW4, the bone width was 0.46 mm at local bone and 0.23 mm at ARP/GBR; and at CW7, the bone width was 0.22 mm at local bone and 0.02 mm at ARP/GBR. There was no discernible difference (P>0.05). Resorption of the alveolar bone width was only noticeable at the middle third of the sites. Long-term alterations in the alveolar bone width surrounding dental implants at local and rebuilt bone sites can be observed using CBCT images.

## Background:

Dental implants are used to replace missing natural teeth. Sufficient bone width around implant site is important for implant success. The eruption of teeth and functional requirements of mastication causes constant and fast remodelling of alveolar bone [1]. Alveolar ridge resorption typically takes place in the first six months following tooth extraction. Bone resorption varies from person to person and in same person at different times [2]. 3.79 mm was the mean amount of ridge resorption over the first six months following tooth extraction, according to a systematic review by Tan *et al.* [3]. As implant patients receive long-term care, bone loss becomes a bigger issue. Clinical examination still faces difficulties in monitoring vertical bone abnormalities surrounding oral or vestibular components of the tooth or implants [4]. Various imaging techniques have been used to evaluate bone volume and dimensions. Radiographic parametric analysis of the mesial and distal bone has been successfully applied to dental implant evaluation. The tissue surrounding the implant, the amount of marginal bone loss, and the state of the implant mechanics component could all be determined by radiographic examination of dental implants [5].Variations in imaging angles or tissue overlap can lead to errors in 2-dimensional (2D) radiography images [6]. Interproximal alveolar bone levels are visible on conventional intra-oral radiographs (CRs). Implant site assessment has been one of the indications for CBCT imaging since its inception in 1998 [4]. Alveolar bone's three-dimensional (3D) form can be measured with CBCT. Additionally, it is capable of measuring buccal alveolar bone with a high degree of precision [2]. The identification and treatment of peri-implant bone abnormalities are improved by the use of CBCT [7]. CBCT was able to correct the image distortion and magnification that came with older imaging methods. In CBCT, linear measurement accuracy was improved with a lower mean error (0.1-0.20 mm). In contrast to CBCT, panoramic distortion could display a high percentage (20%). Compared to CBCT, the radiation exposure from CT is three to ten times higher [8]. These days, CBCT is a crucial diagnostic technique that provides good spatial resolution for dental implant treatment planning [9, 10]. Therefore, it is of interest to examine changes in alveolar bone width surrounding dental implants at natural and rebuilt bone locations using CBCT.

## Materials and Methods:

The present study was conducted on 60 participants of both genders, who received dental implants after considering inclusion and exclusion criteria. The study was done after obtaining ethical consent from institutional ethics bard and informed consent from all the participants. Every participant's name, age, gender, and other details were recorded. A comprehensive oral examination was performed on each patient, and then the implant recipient site was scanned using CBCT technology. Using the CBCT i-CAT scan and Blue sky Plan® software, the distal, mesial, lingual and buccal bone levels surrounding the dental implants were assessed in this study. All conventional surgical procedures were followed for inserting dental implants. All patients had measurements of the horizontal alveolar bone widths around implants at 3 levels: subcrestal width 1 mm (CW1), subcrestal width 4 mm (CW4), and subcrestal width 7 mm (CW7). The collected data were statistically analysed, with a P value of less than 0.05 being regarded as significant.

## Results:

There were 32 male patients and 28 female patients out of 60 totals. The mean bone width was 7.02 mm at CW1 prior to surgery and 6.91 mm afterward; it was 8.52 mm at CW4 and 8.13 mm afterward; and it was 10.21 mm at CW7 prior to surgery and 10.08 mm afterward. The difference was significant (P< 0.05) (Table 1). At CW1, the bone width was 0.38 mm at native bone and -0.02 mm at ARP/GBR; at CW4, the bone width was 0.46 mm at native bone and 0.23 mm at ARP/GBR; and at CW7, the bone width was 0.22 mm at native bone and 0.02 mm at ARP/GBR. The difference was not statistically significant (P>0.05) (Table 2).

## Discussion:

Dental implants require enough buccal bone support in order to be stable both initially and over time. For the implant to be supported circumferentially there must be sufficient bone thickness [11]. Using CBCT, we examined variations in alveolar width surrounding dental implants at natural and rebuilt bones. We observed a substantial shift in bone width by comparing CBCT scans obtained prior to and during implant operation. After loading, bone levels were measured six and twelve months later. The twenty dental implants that were implanted had bone loss surrounding them, according to the CBCT measures of bone level. Dehiscence problems at the implant platform are frequently caused by the uneven association at the bone crest and the resorption of the bony width at the coronal side. This results in scattering in CBCT images and impairs the measurement of the alveolar bone width at the coronal third [2]. Studies by Uraz *et al.* and Sasada *et al.* have documented some bone loss at the crest level surrounding dental implants [12, 13]. Using CBCT, Rawat *et al.* evaluated alterations in the alveolar bone width surrounding dental implants at natural and rebuilt bone locations. They came to the conclusion that only the middle third of all sites showed evidence of considerable alveolar bone width resorption [4]. Our findings are consistent with these results. After a year of typical loading, Payne *et al.* observed a 0.35 mm loss in bone height at the crest; after two years, the bone loss was just 0.09 mm [10]. According to Al-Jaboori *et al.*'s r esearch, bone thickness at the coronal levels is lower and more prone to resorption than at the apical sections [8]. According to Block MS *et al.*'s conclusion, the buccal bone's thickness appears to be preserved over time, irrespective of the technique employed [14]. Before and after implant surgery, Kai-Fang Hu *et al.* measured changes in the alveolar bone width surrounding dental implants at native and reconstructed bone locations. They discovered no appreciable differences in implant-peripheral bone width between native and rebuilt bone sites, which run counter to our findings [2]. Kolte colleagues used cone-beam computed tomography (CBCT) to measure the buccal and lingual bone width in posterior dentate and edentulous sites. They came to the conclusion that crestal bone width (CBW) was lower at edentulous sites than at apical levels. There are clear consequences for implant treatments because the coronal bone width on the buccal and lingual sides of dentate sites is smaller than the apical level [15].

Al-Fakeh *et al.* evaluated changes in bone thickness and density in the maxillary and mandibular jaws after insertion of dental implants using CBCT. They came to the conclusion that only at the crestal third there was a substantial decrease in bone thickness [7]. According to Isoda *et al.* there was a substantial correlation between the primary implant's stability and the measured bone quality, and they suggested that the test's evaluation of bone density might be used to forecast the stability of the implant [1]. Cone beam computed tomography is a trustworthy imaging technique to assess pre-surgical immediate implant locations and to gauge alveolar bone thickness, according to Vyas *et al.* conclusion [11]. Dwingadi *et al.* evaluated the results of dental implant therapy by employing CBCT to assess bone problems. They came to the conclusion that CBCT should be a part of every treatment strategy for patients with intermediate to advanced conditions [5].

Determining the size of the remaining alveolar bone is a crucial prerequisite for the successful placement of dental implants [17]. The functional stress can be distributed by maintaining crestal bone [18]. The functional and aesthetic results of prosthodontic restorations are significantly influenced by the reactions of the alveolar bone following implant implantation. A good quality image for measuring the thickness of the buccal and lingual bone plates can be obtained using cone-beam computed tomography [7]. As 2D pictures, the bitewing and periapical radiographs are unable to accurately depict variations in breadth. CBCT measures changes in width on the buccal and lingual sides and offers a three-dimensional picture of an anatomical structure in respect to the teeth and implants. The study's limited sample size is one of its limitations.

## Conclusion:

We found bone resorption in the middle third of the alveolar bone around the implant sites. CBCT scans are useful to assess the alveolar bone width changes surrounding dental implants at native and reconstructed bone sites.

## Figures and Tables

**Table 1:** Evaluation of bone widths around implants before and after the implant placement (in mm)

**Sub-crestal levels**	**Pre- surgery**	**Post-surgery**	**P value**
CW1 (crestal level)	7.02	6.91	0.47
CW4 (middle level)	8.52	8.13	0.05
CW7 (Basal level)	10.21	10.08	0.52

**Table 2:** Alteration of bone width around implants at native bones and reconstructed bone (ARP/GBR) in mm

**Sub-crestal levels**	**Native bone**	**ARP/GBR**	**P value**
CW1	0.38	-0.02	0.04
CW4	0.46	0.23	0.15
CW7	0.22	0.02	0.28
